# Rhode Island Medical Society

**Published:** 1871-02

**Authors:** 


### RHODE ISLAND MEDICAL SOCIETY.

At the quarterly meeting of the Rhode Island Medical Society, on Wednesday, Dec. 21st, Dr. L. F. C. Garvin read an essay upon "Alcohol, considered as a Medicine and a Nutriment."

Dr. Garvin stated as the result of his experience in practice, that it was rarely beneficial to his patients, that it could not be considered a food, and that it simply arrested a waste of the tissues. He thought it should be classed with other poisonous drugs, and its sale restricted, like those, to druggists and apothecaries, and that it was the duty of every censcientious physician to teach the young it was a potent poison, and seek to banish its use from the homes he was called to visit It was an able paper, and a strong argument against the use of alcohol as a remedy for any disease.—*Medical and Surgical Journal.*

